# New York Journal of Medicine

**Published:** 1845-04

**Authors:** 


### NEW YORK JOURNAL OF MEDICINE.

This Journal is now under the editorial care of Charles A. Lee, M.D., professor of Pathology and Materia Medica in Geneva College, and favorably known to the profession as editor of several works, to which he has made valuable additions. Dr. Lee is a clear, instructive writer, and discriminating critic, and has the industry and independence requisite to form an able editor. In his hands the New York Journal will maintain the respectable rank to which it rose under the management of the lamented Forry.

